# Cross-Talk Between Extracellular Matrix and Skeletal Muscle: Implications for Myopathies

**DOI:** 10.3389/fphar.2020.00142

**Published:** 2020-02-28

**Authors:** Khurshid Ahmad, Sibhghatulla Shaikh, Syed Sayeed Ahmad, Eun Ju Lee, Inho Choi

**Affiliations:** Department of Medical Biotechnology, Yeungnam University, Gyeongsan, South Korea

**Keywords:** collagen, extracellular matrix, laminin, myopathy, skeletal muscle

## Abstract

Skeletal muscle (SM) comprises around 40% of total body weight and is among the most important plastic tissues, as it supports skeletal development, controls body temperature, and manages glucose levels. Extracellular matrix (ECM) maintains the integrity of SM, enables biochemical signaling, provides structural support, and plays a vital role during myogenesis. Several human diseases are coupled with dysfunctions of the ECM, and several ECM components are involved in disease pathologies that affect almost all organ systems. Thus, mutations in ECM genes that encode proteins and their transmembrane receptors can result in diverse SM diseases, a large proportion of which are types of fibrosis and muscular dystrophy. In this review, we present major ECM components of SMs related to muscle-associated diseases, and discuss two major ECM myopathies, namely, collagen myopathy and laminin myopathies, and their therapeutic managements. A comprehensive understanding of the mechanisms underlying these ECM-related myopathies would undoubtedly aid the discovery of novel treatments for these devastating diseases.

## Introduction

Skeletal muscle (SM) is a contractile tissue primarily comprised of multinucleated myofibers. SM is one of the most important plastic tissues in the human body and accounts for around 40% of total body weight (Campbell and Stull, 2003; Frontera and Ochala, 2015; Kim et al., 2019). SMs contain multipotent precursor cells called muscle satellite cells (MSCs), which are localized below the basal lamina (BL) in myofibers, play vital roles in maintaining the integrity of SM, and participate in muscles regeneration *via* an organized myogenic program (Asakura et al., 2001). After injury, MSCs activate, proliferate, and fuse to form myofibers, which constitute the functional contractile parts of mature SM (Carlson and Faulkner, 1983; Bonilla et al., 1988; Stuelsatz et al., 2017). The developmental process of multinucleated myofibers with contractile capability from MSCs is termed myogenesis, and involves cell cycle arrest, cell fusion, increases in nuclear sizes, and the peripheral localization of nuclei (Charge and Rudnicki, 2004). Myogenesis is a decidedly regulated mechanism that is determined by the co-expressions of Pax3, Pax7, and myogenic-regulatory factors such as Myf5, Mrf4, MyoD, and myogenin in MSCs (Zammit and Beauchamp, 2001; Relaix et al., 2005; Baig et al., 2019; Kim et al., 2019; Lee et al., 2019).

**Figure 1:**
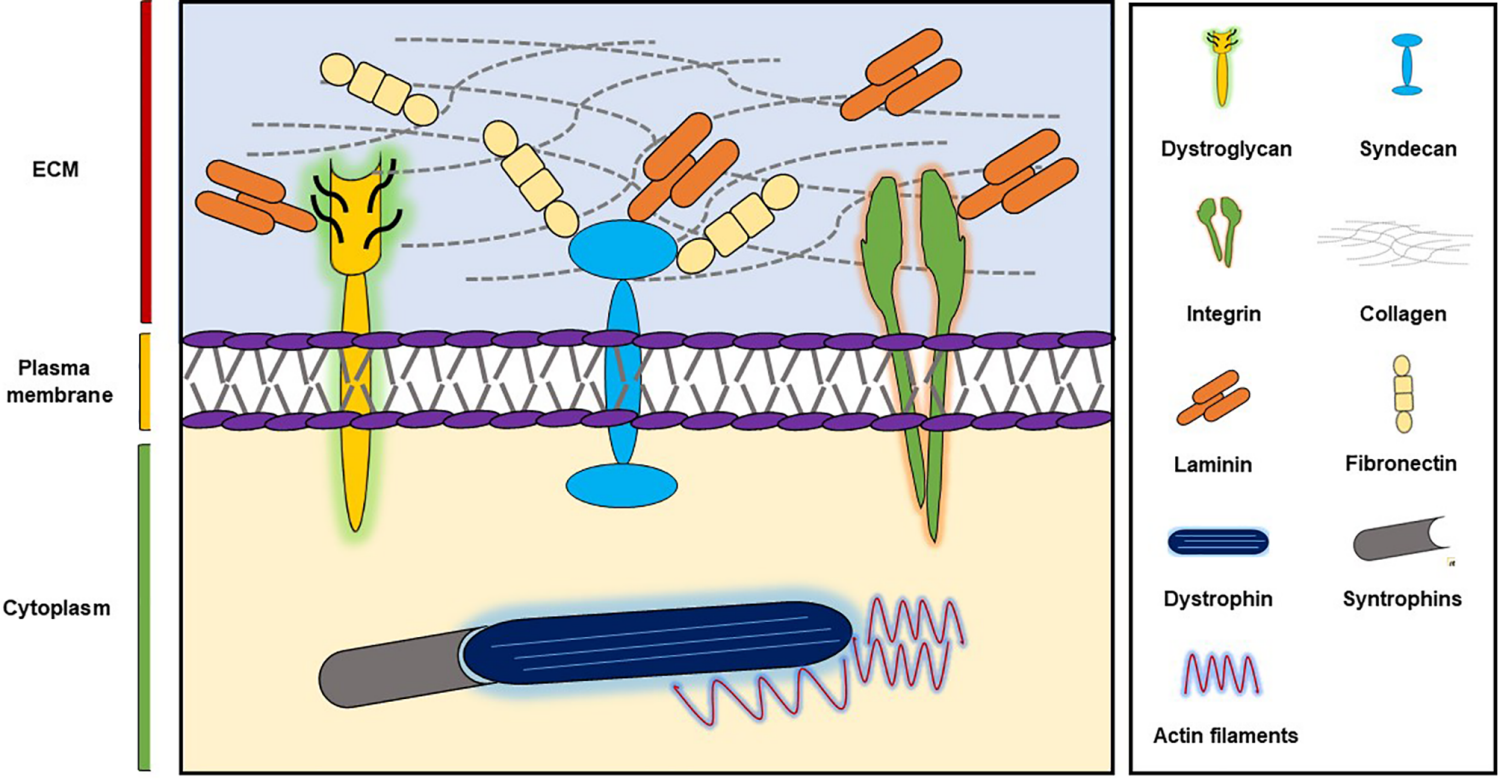
ECM components and their major receptors.

SM supports skeletal development, aids skeletal movement, controls body temperature, and manages glucose uptake (Ahmad et al., 2018) and is composed of large numbers of long, multinucleated filaments, which are organized by extracellular matrix (ECM) (Davis et al., 2013). ECM plays important roles during wound healing, embryogenesis, and tissue repair and provides integrity and biochemical signals to cells (Govindan and Iovine, 2015; Ahmad et al., 2018) and is composed of glycoproteins like collagens, fibronectin (FN), and laminins (Frantz et al., 2010) (**Figure 1**). Furthermore, two major SM ECM proteins, that is, collagen and laminin, are known to be associated with myopathies. In previous studies, we explored the roles of a small number of ECM proteins like fibromodulin, matrix gla proteins, and dermatopontin during different stages of myogenesis (Lee et al., 2016; Ahmad et al., 2017; Lee et al., 2018; Kim et al., 2019) and found fibromodulin, as well as dermatopontin, is involved in the vigorous recruitment of MSCs at sites of injury, and thus, aids SM regeneration (Lee et al., 2018; Kim et al., 2019).

Secreted elements, such as diverse kinds of growth factors, are released during SM repair and are used to monitor muscle regeneration, but their roles and impacts on SM remodeling remain obscure (Karalaki et al., 2009). The transmembrane receptors send signals into cells from the ECM, and thus, initiate several cell functions, for instance survival, development, movement, and differentiation, which are important for sustaining the homeostasis (Theocharis et al., 2016). Notably, SM fibrosis arises in diabetes and muscular dystrophies and during immobilization and aging (Alnaqeeb et al., 1984; Williams and Goldspink, 1984; Berria et al., 2006; Gillies and Lieber, 2011).

In this review, we briefly describe SM and its major ECM components with emphasis on their roles in muscle-related diseases, which are generally fibrotic diseases or types of muscular dystrophy. We believe an in-depth understanding of the underlying mechanisms of ECM-related myopathies will aid the discovery of novel therapeutic options for the management of these devastating diseases.

### ECM Role in the Development and Function of SM

ECM is a well-organized non-cellular environment that undergoes regular cycles of alterations, degradation, as well as reassembly. Cell-matrix communication contribute a vital role in cell adhesion and migration, and therefore, is critically important during embryonic and adult myogenesis (Goody et al., 2015). Various ECM proteins (e.g., dermatopontin, nidogen/entactin, periostin, and osteopontin) contribute to the regulations of cell-matrix communications and matrix assembly (Funanage et al., 1992; Norris et al., 2007; Kato et al., 2011; Kim et al., 2019).

Myoblasts fuse to form SM fibers during the fetal stage, and myofiber numbers remain constant throughout the postnatal period. However, during this period, myofiber sizes are increased by MSC fusion. SM is among the most adaptive of body tissues, and its regenerative ability after SM injury depends on MSCs, which though generally quiescent, turn on and start to proliferate, differentiate to myoblasts, and then fused with myofibers to reestablish the contractile nature of SM after SM or MSC injury (Griffin et al., 2010).

Cross-talk between MSCs and their microenvironments determines SM regeneration. According to the MSC niche concept, the fates of stem cells are determined by stimuli arising from surrounding environments. Furthermore, MSCs exist in specific niches, consisting of muscle myofibers, muscle residence cells, vascular systems and ECM (Wilschut et al., 2010), and their functions are profoundly influenced by different microenvironments.

MSCs are enclosed in laminin and are found between muscle fibers and BL, the latter of which is composed of collagen IV and laminin networks. MSCs bind to these components using integrin receptors (Sanes, 2003), which are heterodimeric transmembrane receptors that critically transform extracellular signals into intracellular responses and interact with ECM as directed by intracellular changes (Askari et al., 2009). BL also functions as a mechanical barrier that prevents MSC migratory loss from normal SM and it might also be involved in the inhibition of MSC differentiation in the absence of damage (Sanes, 2003).

Almost all myofibers in SM develop from somites, that is, from mesodermal structures that evolve in the early embryonic segmentation (Christ and Ordahl, 1995; Pourquie, 2001). FN and its communication with integrin play vital roles during somatic cell polarization and guidance (Ostrovsky et al., 1983; Lash et al., 1985). Furthermore, decorin has been found to participate in SM development by inhibiting myostatin activity, and thus, enhancing myogenic cell proliferation and differentiation (Kishioka et al., 2008).

### Major Components of Muscle ECM

The ECM is comprised of various proteoglycans and fibrous proteins (e.g., collagens, elastins, FN, and laminins). Collagen is the main structural protein in SM ECM and holds 1 to 10% of SM dry weight (Dransfield, 1977; Gillies and Lieber, 2011). The two main ECM types are: 1) interstitial matrices—connective tissue matrices comprised of mixtures of collagens, elastins, FN, proteoglycans, and glycosaminoglycans (Florin et al., 2004), and 2) pericellular matrices—which interact with cells and have more diverse molecular compositions than surrounding interstitial matrix (Thorsteinsdottir et al., 2011).

ECM represents up to 10% of muscle weight and can be classified as endomysium, perimysium, or epimysium. The endomysium contains individual myofibril, whereas the perimysium partitions SMs into fascicles, and outer support to whole SM is provided by the epimysium (Kjaer, 2004). Type I collagen has been reported to be the predominant perimysial collagen, while type III collagen is dispersed between endomysium and epimysium (Light and Champion, 1984). BMs are predominantly comprised of laminins, collagen type IV, nidogen (entactin), and perlecan (Lebleu et al., 2007), whereas collagen types VI, XV, and XVIII are also present in the BMs of SM (Halfter et al., 1998). Reticular lamina, present beneath BM, is mostly comprised of collagen fibrils (types I, III and VI) and FN in a proteoglycan rich gel (Garg and Boppart, 2016).

Laminin, collagen IV, nidogen/entactin, agrin, biglycan, and perlecan form the BL that surrounds SM fibers (Gullberg et al., 1999; Tunggal et al., 2000; Jimenez-Mallebrera et al., 2005), and it has been proposed laminin in ECM stimulates myoblast proliferation and differentiation (Rooney et al., 2009). Laminin-211 is the predominent laminin isoform in BMs of adult SM (Ehrig et al., 1990; Sasaki et al., 2002), and integrins and non-integrins are two potential groups of laminin-211 receptors. Integrins are αβ heterodimeric transmembrane proteins with a huge number of functions that include adhesion, migration, and differentiation (Hynes, 2002). α7β1 is the major integrin of adult SM (Song et al., 1992; Burkin and Kaufman, 1999), and α-dystroglycan is the primary non-integrin cell surface receptor (Ahmad et al., 2018). The integrins α1β1, α2β2, α3β1, α6β1, α6β4, α7β1, α9β1, αvβ3, and αMβ2 are reported to bind laminin, and most recognize the globular domain of its long arm (Wondimu et al., 2004; Tzu and Marinkovich, 2008; Durbeej, 2010). However, only α3β1, α6β1, α6β4 and α7β1 integrin are considered highly selective laminin receptors (Humphries et al., 2006; Nishiuchi et al., 2006; Barczyk et al., 2010).

Proteoglycans in SM ECM predominantly belong to the small leucine-rich proteoglycan family, and the commonly found proteoglycans in SM ECM are chondroitin sulfate and dermatan sulfate glycosaminoglycans (Brandan and Inestrosa, 1987). Furthermore, interactions between proteoglycans and collagen sustain ECM structure and organization. Proteoglycans bind to collagen at particular positions (Pringle and Dodd, 1990), and thus, proteoglycan to collagen ratios vary in ECM. In addition, the leucine-rich repeats of decorin bind with collagen type I to determine the role of decorin as a regulator of collagen fibrillogenesis in SM (Gillies and Lieber, 2011).

### Myopathies and ECM

Myopathy refers to muscle diseases in which muscle weakness due to muscle fiber dysfunction is the primary symptom. Other symptoms include muscle cramps, stiffness (myotonia), and spasm. Myopathies are broadly categorized as inherited and/or acquired (Stenzel and Schoser, 2017). Inherited myopathies predominantly affect SM tissues and are generally caused by mutations in the genes responsible for SM development, as exemplified by different types of non-dystrophic and dystrophic SM disorders, which manifest an extensive range of genetic and biochemical features. Muscular dystrophies, congenital metabolic myopathies, and myotonia are the most prevalent inherited myopathies (Cardamone et al., 2008; González-Jamett et al., 2018). Common muscle cramps are categorized as an acquired myopathy, for example; hypothyroid and hyperthyroid myopathies are caused by thyroid gland abnormalities (Ruff and Weissmann, 1988; Sindoni et al., 2016). Other systemic diseases (e.g., endocrine disorders, pituitary or adrenal dysfunction, Cushing's disease, sarcoidosis, diabetes mellitus, mixed connective disease, and electrolyte imbalance) and toxic myopathies caused by medications are also examples of acquired myopathies (Chawla, 2011). Furthermore, SM myopathies have major effects on pathogenesis and clinical outcomes may result in cardiac arrest (Chapleau, 2014).

Several human diseases are associated with ECM abnormalities, and ECM components are implicated in the pathologies of disorders that affect almost every organ system. These ECM-linked diseases are generally attributable to factors ranging from abnormal signaling functions to inadequacies of the structural components of vital organs (Iozzo and Gubbiotti, 2018). Several SM-associated genetic disorders are typically caused by mutations in ECM elements and cell surface receptors. Interestingly, more than 150 ECM proteins have been reported to interact with integrin receptors (Zaidel-Bar et al., 2007; Ahmad et al., 2018).

**Figure 2:**
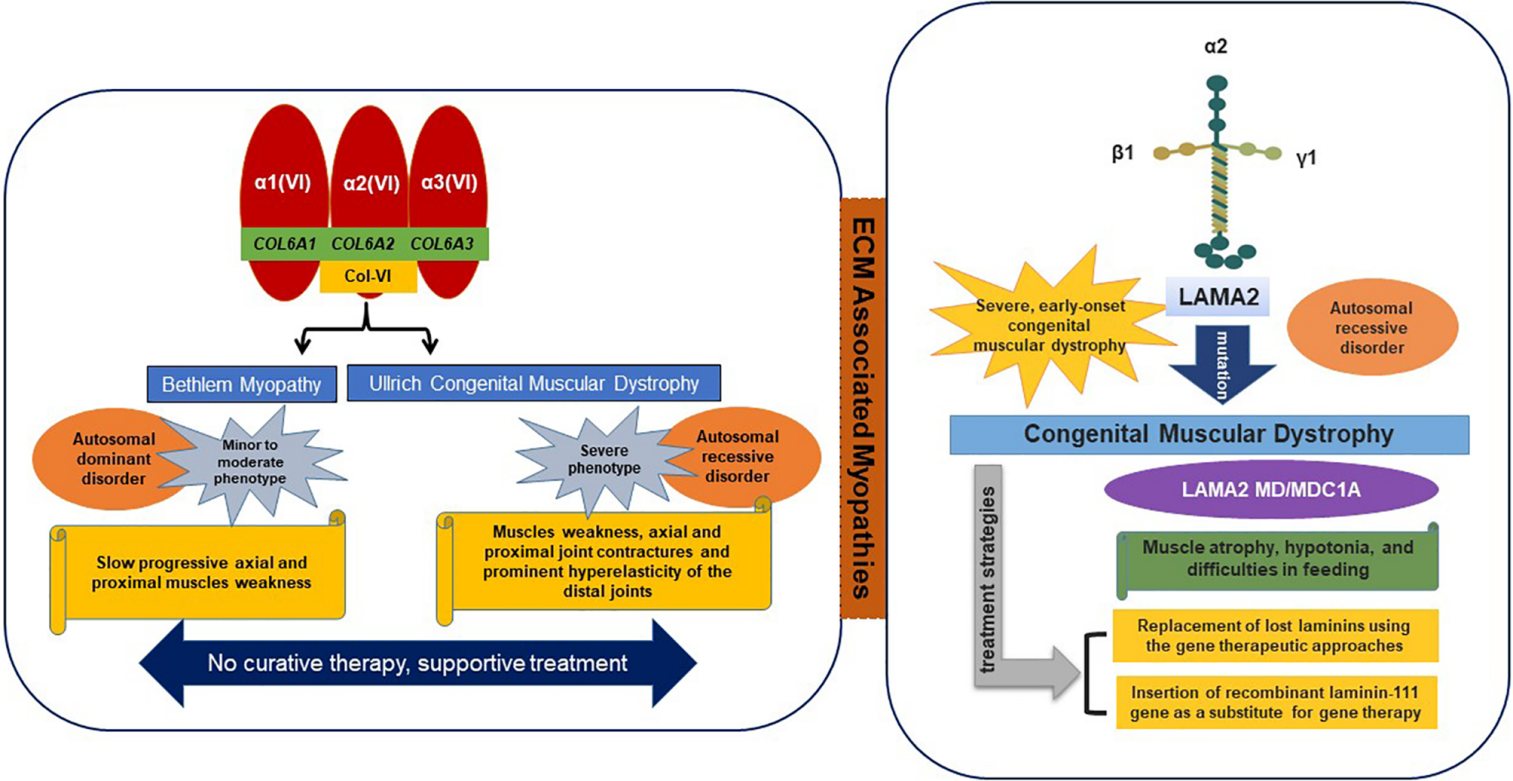
ECM-associated myopathies.

The main ECM transmembrane receptor of SM is composed of dystrophin-glycoprotein complex (DGC), utrophin glycoprotein complex (UGC), and α7β1 integrin complex. These laminin-binding protein complexes can transform the progression of disease, and thus, are viewed as curative targets for disease intervention (Van Ry et al., 2017). α7β1 integrin is a laminin receptor found on the exterior surfaces of skeletal myoblasts and myofibers. In Duchenne muscular dystrophy (DMD), articulation of α7β1-mediated ECM binding may compensate for the absence of the dystrophin-mediated linkage. Collagen and laminin related dystrophies are typical of the diseases associated with these SM receptors (**Figure 2**).

In addition, the downregulation of integrin articulation may add to the progression of congenital laminin deficiencies (Burkin and Kaufman, 1999). Dystrophin is related to a muscle membrane (sarcolemmal) glycoprotein complex, which provides linkage with laminin. Interestingly, in the absence of dystrophin-related proteins (43DAG, 50DAG, 59DAP, 35DAG, and 156DAG) were markedly down-regulated in the sarcolemma of DMD patients and MDX mice (Matsumura et al., 1992).

### Collagen Myopathy

Collagens are the most common and major component of ECM and are reported in almost all connective tissues. They fulfill a number of critical functions in SM including the transmission of forces to bones, tensile strength, and elasticity, and are also involved in the regulations of cell attachment and differentiation. Collagens also play critical roles in cell-to-ECM interactions *via* several transmembrane receptors. Collagen type VI (COL VI) is a component of SM ECM in terms of these interactions (Jobsis et al., 1996).

Collagen VI is consist of α1, α2, and α3 chains, which are encoded by COL6A1 and COL6A2 on chromosome 21q22 and by COL6A3 on 2q37, respectively (Allamand et al., 2011). Mutations in all three of these genes result in two main muscle disorders types, namely; 1) Ullrich congenital muscular dystrophy (UCMD) (severe phenotype) and 2) Bethlem myopathy (minor to moderate phenotype). Recently, two additional phenotypes were found to be associated with mutations in the COL6A2 gene, that is, limb-girdle muscular dystrophy and myosclerosis (an autosomal recessive phenotype) (Bushby et al., 2014). In ECM, collagen VI interacts with various molecules (collagen II, IV, XIV, and decorin) and cell surface receptors (fibulin 2, hyaluronan, membrane-associated chondroitin sulfate proteoglycan 4, and biglycan) (Bonnemann, 2011).

#### Ullrich Congenital Muscular Dystrophy (UCMD)

UCMD is usually defined as an autosomal recessive condition that results in muscle weakness, contractures of proximal joints, and prominent hyperelasticity of distal joints (Lampe and Bushby, 2005). The UCMD phenotype is caused by loss-of-function mutations or dominant missense mutations involving glycine substitutions in the triple helical (Gly-Xaa-Yaa) motif or dominant exon-skipping mutations (Lampe and Bushby, 2005; Lampe et al., 2008). Mutations in the COL6A2 gene and more recently in the COL6A3 gene have been shown to cause UCMD (Demir et al., 2002). Usually, walking is delayed in affected children and they are unable to jump or run properly (Camacho Vanegas et al., 2001).

#### Bethlem Myopathy

Bethlem myopathy is a dominantly congenital, comparatively mild disease caused by mutations in the COL6A1, COL6A2, or COL6A3 genes characterized by progressive proximal muscle weakness and contractures (joint stiffness) of fingers, wrists, elbows, and ankles. Symptoms may be observed before birth due to reduced fetal movement, in early childhood due to late motor skill development, and in adults due to contractures of Achilles tendons or fingers (Lampe and Bushby, 2005; Baker et al., 2007).

The benchmark for the diagnosis of collagen myopathies is the identification of mutations in the COL6A1, COL6A2, or COL6A3 genes. Diagnoses of UCMD and Bethlem myopathy generally depend on distinctive clinical topographies in combination with mildly increased or normal levels of serum creatine kinase. Muscle biopsy is used to differentiate Bethlem myopathy and UCMD, for example, in Bethlem myopathy collagen VI immunolabeling of BL and endomysium are typically normal but in UCMD they are absent to obviously reduced (Lampe and Bushby, 2005). Treatments of Bethlem myopathy and UCMD involve supportive care following identical philosophies based on considerations of age at onset and observed symptom severity (Allamand et al., 2011).

Obstructive lung disease is common in patients with Bethlem myopathy and carries the risk of subsequent respiratory inadequacy (Bonnemann, 2011).

### Laminin Myopathy

The laminin-α2 subunit is encoded by the LAMA2 gene and is primarily expressed in SM, and laminin-211 is the most abundant isoform found in BMs. Mutations in LAMA2 result in the most common types of congenital muscular dystrophies, that is, LAMA2 MD or MDC1A (Congenital type 1A), which are characterized by disruption of laminin-211 and account for 10–30% of reported cases (Sframeli et al., 2017; Mohassel et al., 2018). In general, the common symptoms of laminin myopathy are hypotonia, muscle weakness, and feeding difficulties, though joint contractures, including contractures of fingers and ankles, typically develop in later stage disease. The therapeutic strategies for LAMA 2 MD include replacement of lost laminins using gene therapeutic approaches or the administration of recombinant laminin-111 (Yurchenco et al., 2018). In addition to binding with typical receptors (integrins and dystroglycans), laminins bind with other ECM macromolecules such as nidogens and perlecan (Carmignac and Durbeej, 2012).

## Concluding Remarks

SM is greatly influenced by ECM composition, and collagen is one of the main structural proteins in ECM. ECM myopathy is a group of genetic disorders caused by mutations in genes encoding proteins that provide critical associations between the ECM and muscle cells, and UCMD and Bethlem myopathy are the main SM diseases caused by mutation of collagen VI. Laminin largely composes the BL that surrounds muscle fibers, and laminin-211 plays a crucial role in SM function. LAMA2 MD and MDC1A are destructive muscular dystrophies caused by laminin α2 chain loss and have no cure. The discovery of additional information related to collagen VI and laminin 211 in human SM may prove to be decisive in terms of the developments of future therapies. The objective of this article was to improve understanding by exploring relations between ECM components and related diseases so as to aid the development of effective treatments. We recommend further research work be conducted to characterize ECM-related myopathies more precisely.

## Author Contributions

KA, SS, and SA wrote the initial draft of the paper and designed the figures. EL contributed to the systematic review of the literature. IC critically analyzed and approved the paper.

## Funding

This research was supported by the National Research Foundation of Korea (NRF) funded by the Korean government (MSIP: Grant No. NRF-2018R1A2B6001020) and a grant from the Next-Generation BioGreen 21 Program (Project No. PJ01324701), Rural Development Administration, Republic of Korea.

## Conflict of Interest

The authors declare that the research was conducted in the absence of any commercial or financial relationships that could be construed as a potential conflict of interest.

## Abbreviations

SM, Skeletal muscle; ECM, Extracellular matrix; MSCs, Muscle satellite cells; BL, Basal lamina; FN, Fibronectin; DGC, Dystrophin glycoprotein complex; UGC, Utrophin glycoprotein complex; DMD, Duchenne muscular dystrophy; BM, Basement membrane; UCMD, Ullrich congenital muscular dystrophy.
